# Supplementary material for: Climate change and tree cover loss affect the habitat suitability of Cedrela angustifolia: evaluating climate vulnerability and conservation in Andean montane forests
Source: PeerJ. 2025 Feb 27;13:e18799. doi: 10.7717/peerj.18799 (PMC11874945; doi:10.7717/peerj.18799)
Supplement: Supplemental Information 1 [file peerj-13-18799-s001.docx]

Table S1: ODMAP protocol for reporting *Cedrela angustifolia* distribution models following Zurell et al. (2020).

| **ODMAP elements** | **Contents** |
| --- | --- |
| **OVERVIEW** | |
| Authorship | - Authors: Fressia N. Ames-Martínez, Iván Capcha, Anthony Guerra, Janet Inga, Harold R. Quispe-Melgar, Esteban Galeano, Ernesto C. Rodríguez-Ramírez. - Contact email: [fressiames@gmail.com](mailto:fressiames@gmail.com)   Title: How do climate change and tree cover loss affect the habitat suitability of *Cedrela angustifolia*? Evaluating climate vulnerability and conservation in Andean Montane Forests |
| Model objective | Model objective: Mapping and interpolation.  Target output: Suitability habitat index (probability of species presence). |
| Taxon | *Cedrela angustifolia*, plants. |
| Location | Ecuador, Peru, Bolivia, Argentina; South America. |
| Scale of analysis | Spatial extent: -28.311835, -78.151898, -65.871586 -0.590849 (xmin, xmax, ymin, ymax), covering ~3,900 km.  Spatial resolution: ~1 km x 1 km.  Temporal extent: present and future (2040; 2070; 2100 years).  Boundary: natural, political. |
| Biodiversity data overview | - Observation type: standardized monitoring. - Response/Data type: presence-only. |
| Type of predictors | Climatic, habitat. |
| Conceptual model/Hypotheses | We hypothesized that the most suitable habitat for *C. angustifolia* would be negatively affected by climate change and tree cover loss by 2100 |
| Assumptions | The present and future *Cedrela angustifolia* distribution is not at equilibrium with the environment and is mostly driven by climate. There is no sampling bias in the species occurrence data.   - Relevant ecological drivers (or proxies) of species distributions are comprised. - Species are at (pseudo-) equilibrium with their environment. - Species show similar responses to the bait and the sampling instrument. - Sampling is adequate and representative (and any biases are accounted for/corrected). |
| SDM algorithms | - Modeling techniques: MaxEnt and Random Forest - Model complexity: MaxEnt models were built with linear, quadratic, product, threshold, and hinge features. - Ensembles: We combined model types to form ensemble predictions, we used KUENM package for model selections. |
| Model workflow | We got 104 *Cedrela angustifolia* occurrence records from South America, and fitted SDMs using the KUENM package (Cobos et al. 2019) with MaxEnt algorithm and one candidate predictor set (environmental).  Model calibration was done with careful consideration of data limitations, and the effects of various assumptions available were assessed via experimentation.  We combined model predictions into ensembles using a weighted KUENM algorithm. We evaluated predictive performance using AUC, Partial ROC (Receiver Operating Characteristic), omission rate, and the optimal complexity parameter. We then calculated predicted range sizes from the binary maps and range sizes from the environmental-only, and suitability habitat we calculated with climate, land cover and protected natural areas. |
| Software, codes, and data | - Software: Maxent 3.4.1 and R version 4.1.3, packages ‘raster’, ‘rgdal’, ‘KUENM’, ‘fuzzySim’, ‘virtualspecies’,’RandomForest’ and ‘sdm’. - Data availability: Data is available in GBIF secretariat (2022), Missouri Botanical Garden (2022), Pennington and Muellner (2010) and Muellner et al. (2012). |
| **DATA** | |
| Biodiversity data | - Taxon names: *Cedrela angustifolia.* - Taxonomic reference system: We followed the updated list of the International Plant Name Index (https://www.ipni.org/n/ 577723-1) and Tropicos for Missouri Botanical Garden (https://tropicos.org/name/ 20400040). - Ecological level: species. - Biodiversity data source: We derived data from literature sources, including our earlier compilation (Pennington and Muellner, 2010; and Muellner et al., 2012). As an independent source, we conducted fieldwork studies on AMFs from 2019 and we complemented this collected information with satellite imagery observation. - Sampling design: 104 occurrence points of *Cedrela angustifolia*. - Background data: Random, as no information on the sampling process was available. - Clipping: Andean Montane Forest from South America. - Cleaning: We removed from our data: i) duplicate occurrence points; and ii) imprecise point occurrences (e.g. coordinates assigned to municipalities). - Details on potential errors and biases: 10,000 randomly generated background points for MaxEnt. |
| Data partitioning | We conducted 100 replicate analyses for *Cedrela angustifolia* based on a 50% bootstrap of occurrence data. |
| Predictor variables | - Predictor variables: - Present: elevation data, 19 bioclimatic variables, soil data, tree cover loss data and NDVI. - Future: 19 bioclimatic variables for the five models for future climate projections (GFDL-ESM4, IPSL-CM6A-LR, MPI-ESM1-2-HR, MRI-ESM2-0, and UKESM1-0-LL), selected two future climate scenarios (Shared Socioeconomic Pathways; SSP 3-7.0 and 5-8.5). - Data sources: We downloaded elevation data and bioclimatic variables from CHELSA database (<http://chelsa-climate.org/>; Karger et al. 2022), soil grids, global forest watch and earth explorer. - Spatial extent: bioclimatic and elevation(Worldwide). - Spatial resolution: environmental variables (30 arc-second or ~1 km spatial resolution). - Temporal extent: present (1980-2010) and future (2040-2070). - Map projection: WGS 1984 - Dimension reduction: We reduced dimension with the False Discovery Rate calculation (FDR), the multicollinearity degree (*r* <0.7), coefficient of determination of linear regression, tolerance, and the variance inflation factor (VIF), the Bayesian information criterion (BIC), Akaike (AIC), Jackknife analysis, and our knowledge of *Cedrela angustifolia* responses to specific environmental factors. |
| **MODEL** | |
| Multicollinearity | We performed a Pearson’s correlation test to check for multicollinearity among the environmental variables. |
| Model settings | Maxent: default from KUENM package (Cobos et al. 2019) that uses presence-only data and 10,000 randomly generated background points. |
| Model estimates | We used the determination coefficient of linear regression, tolerance, and the variance inflation factor (VIF), the Bayesian information criterion (BIC), Akaike (AIC), and Jackknife analysis. |
| Model selection | We used the average performance evaluation indicators (AUC), Partial ROC (Receiver Operating Characteristic), omission rate, and the optimal complexity parameter (AIC-Akaike Information Criterion). |
| Non-independence correction/analysis | None. |
| Threshold selection | We used a logistic threshold of training presence clipping, which corresponds to the 10% of data with the lowest probability value, commonly used in conservation studies (Radosavljevic and Anderson, 2014), and we created a probability map from each modeling output. |
| **ASSESSMENT** | |
| Performance statistics | - Model evaluation (AUC with 95%-CI, partial ROC, omission rate, and optimal complexity parameter-ΔAIC). - Model validation by bootstrapping with 10000 iterations (AUC, calibration plot). We checked for spatial autocorrelation (correlograms). |
| Plausibility check | We checked model plausibility by assessing partial dependence plots. |
| **PREDICTION** | |
| Prediction output | We predicted the present and future potential *Cedrela angustifolia* presence.  We included 10,000 bias files and environmental variables to assess potential habitat suitability analysis. We performed a Gaussian Kernel with QGIS software to avoid the sampling bias effect and identify the highest potential suitability areas, to visualize the areas that are congruent between the predicted models and suitability areas. |
| Uncertainty quantification | N/A |
|  |  |
