# Supplementary material for: Climate change and tree cover loss affect the habitat suitability of Cedrela angustifolia: evaluating climate vulnerability and conservation in Andean montane forests
Source: PeerJ. 2025 Feb 27;13:e18799. doi: 10.7717/peerj.18799 (PMC11874945; doi:10.7717/peerj.18799)
Supplement: Supplemental Information 2 [file peerj-13-18799-s002.docx]

Table S2. Mathematical indicators of each chosen best model.

| Model | Parameters | Mean AUC ratio | Partial ROC | Omission rate at 5% | AICc | Delta AICc | W AICc |
| --- | --- | --- | --- | --- | --- | --- | --- |
| Present_1_F_lqh_Set13 | Features: Linear, quadratic, hinge.  Regulation multiplier:1  Variables set:13 | 0.9835 | 0.000 | 0.000 | 537.1 | 0.016 | 0.002 |
| UKESM-1-2040_1_F_lqh_SSP370_Set57 | Features: Linear, quadratic, hinge.  Regulation multiplier:1  Variables set:57 | 0.97415 | 0.000 | 0.000 | 547.94 | 0.023 | 0.015 |
| IPSL-cm6a-lr-2040_1.5_F_lqh_SSP585_Set48 | Features: Linear, quadratic, hinge.  Regulation multiplier:1  Variables set:48 | 0.96875 | 0.000 | 0.000 | 549.71 | 0.024 | 0.009 |
| IPSL-cm6a-lr-2070_1_F_lqh_SSP370_Set46 | Features: Linear, quadratic, hinge.  Regulation multiplier:1  Variables set:46 | 0.9865 | 0.000 | 0.000 | 540.72 | 0.020 | 0.006 |
| ESM4-2070_1_F_lqh_SSP585_Set67 | Features: Linear, quadratic, hinge.  Regulation multiplier:1  Variables set:67 | 0.9807 | 0.000 | 0.000 | 538.4 | 0.016 | 0.005 |
| UKESM1-0-ll-2100_1_F_lqh_SSP370_Set26 | Features: Linear, quadratic, hinge.  Regulation multiplier:1  Variables set:26 | 0.9951 | 0.000 | 0.000 | 547.8 | 0.013 | 0.004 |
| UKESM1-0-ll-2100_1_F_lqh_SSP585_Set84 | Features: Linear, quadratic, hinge.  Regulation multiplier:1  Variables set:84 | 0.9924 | 0.000 | 0.000 | 535.7 | 0.017 | 0.002 |
